# Government service delivery and citizen satisfaction in Gauteng, South Africa

**DOI:** 10.3389/fsoc.2026.1779853

**Published:** 2026-04-30

**Authors:** Godswill Nwabuisi Osuafor, Salmon Likoko, Monica Ewomazino Akokuwebe

**Affiliations:** 1 Department of Social Sciences, Faculty of Humanities, Social Sciences & Law, Walter Sisulu University, Mthatha, South Africa; 2 Nigerian British University, Aba, Nigeria; 3 Demography and Population Studies, School of Social Sciences, University of Witwatersrand, Johannesburg, South Africa; 4 SAMRC/Wits Ageing African Adult Research Unit, Department of Paediatrics, School of Clinical Medicine, Faculty of Health Sciences, University of the Witwatersrand, Johannesburg, South Africa

**Keywords:** Batho Pele principles, citizen satisfaction, Gauteng, government service delivery, institutional trust, socio-demographic factors, South Africa

## Abstract

**Introduction:**

Government service delivery is a critical determinant of citizen trust and satisfaction in South Africa, where inequalities and governance challenges shape perceptions of state performance. Gauteng, the country’s most populous and economically dynamic province, provides a revealing case for examining how socio-demographic factors, service experiences, and institutional trust influence satisfaction with provincial, municipal, and ward-level governance. This study examines the patterns of satisfaction and dissatisfaction across tiers of government, highlighting the role of service quality, civic participation, and leadership credibility in shaping confidence.

**Method:**

Data were drawn from Rounds 5 (2017/18), 6 (2020/21), and 7 (2023/24) of the GCRO QoL Survey, a regionally representative study conducted biennially in Gauteng Province. The analytic sample comprised 9,946 respondents aged 18 years and above for each governance level: provincial government, local municipality, and ward councillor. Respondents were categorised into satisfied and dissatisfied groups. Analyses employed descriptive statistics, bivariate tests, and logistic regression to identify predictors of satisfaction.

**Results:**

Socio-demographic, civic, and perceptual factors significantly predicted satisfaction across governance tiers. Older adults, particularly those aged 60+, reported higher satisfaction, while younger cohorts were consistently more dissatisfied. Population group differences were notable: White respondents expressed substantially higher satisfaction at municipal (OR = 1.67) and ward levels (OR = 4.28), with Indian/Asian respondents also more satisfied at the ward level (OR = 2.09). Education had mixed effects: it reduced provincial satisfaction but increased it at municipal and ward levels. Employment status mattered locally, with unemployed individuals reporting lower satisfaction. Civic engagement, including voter registration and awareness of Batho Pele principles, increased the odds of satisfaction. In contrast, crime victimisation, bribery, and neighbourhood protests reduced service quality; however, satisfaction, particularly in sanitation and healthcare, strongly improved.

**Conclusion:**

The study reveals widespread dissatisfaction with government performance in Gauteng, intensified during and after COVID-19, particularly at provincial and municipal levels, with modest recovery at the ward level. Distrust in leadership emerged as the most potent negative predictor across all tiers. These findings underscore the need for institutional reform, equitable service delivery, stronger accountability, and participatory governance to rebuild trust and enhance citizen satisfaction in South Africa.

## Introduction

South Africa has undergone diverse phases of public service delivery since the establishment of democratic governance in 1994. The South African government has delivered several services at the grassroots level to meet citizens’ expectations and promote satisfaction. This aligns with its mission of effective public management and the core principles of good governance (Lamsal and Gupta, 2022; Akokuwebe et al., 2023a). Public satisfaction with government performance has emerged as a pivotal sociological indicator of democratic legitimacy and institutional trust. This applies across a range of political systems, from consolidated democracies to those in democratic transition. Evidence from international research affirms that effective governance is a key determinant of life satisfaction (Akokuwebe et al., 2023b). According to Flavin (2024), well-being across Organisation for Economic Co-operation and Development (OECD) countries is more strongly influenced by government responsiveness and policy outcomes than by electoral participation alone. Similarly, Helliwell et al. (2018) showed that citizens in countries with high institutional trust and low corruption report significantly better mental health outcomes. Ciziceno and Travaglino (2019) also reported greater civic optimism. In the European context, Ott (2011) highlighted that the technical quality of governance, characterised by transparency, effective policy implementation, and fairness, has a stronger relationship with life satisfaction than democratic procedures alone. These findings reflect broader trends in Gauteng and across Africa, where dissatisfaction arises not only from service delivery gaps but also from perceived corruption, weak accountability, and erosion of civic trust (Akokuwebe et al., 2023a; Zhou and Utete, 2023).

Public satisfaction with government performance has shown low records in Gauteng, the most populous and economically significant province in South Africa [Gauteng City-Region Observatory (GCRO), 2023–2024]. The Gauteng City-Region Observatory (GCRO) conducted a Quality of Life Survey between 2017 and 2024. Only 22% of the population reported satisfaction with the performance of national, provincial, or local governments [Gauteng City-Region Observatory (GCRO), 2023–2024]. The QoL index has recorded its lowest satisfaction score since its inception [Gauteng City-Region Observatory (GCRO), 2023–2024]. This decline in public trust highlights long standing issues, including recurring service delivery inadequacies and widespread concerns about corruption. It also underscores the crucial need for governmental accountability (Schoeman and Chakwizira, 2023; Akokuwebe et al., 2025). With its unique mix of urban and peri-urban populations, Gauteng is the nation’s economic powerhouse and a major influence on the socio-political dynamics of the country [Gauteng City-Region Observatory (GCRO), 2023–2024]. The deterioration in public trust in institutions goes beyond a statistical decline and reflects a serious structural problem. It may jeopardise democratic participation and erode social cohesion throughout Gauteng province [Gauteng City-Region Observatory (GCRO), 2023–2024]. Reduced trust in government ability and intentions may lead to social disintegration, civic disengagement, and indifference. However, these challenges are not insurmountable. By addressing these issues, the government can positively impact the general well-being and contentment of the population.

Drawing on comparative views from nations such as Nigeria, Kenya, Ghana, and Mozambique, this study situates Gauteng’s governance issues within a broader African context (Adeyinka and Adewumi, 2023; Ang’anyo Onyango et al., 2024; Xatse and Naong, 2025). The provision of public services throughout the continent has been a recurring source of conflict and, subsequently, a serious issue that has an immediate effect on individuals’ lives. With over 7,000 youth-led protests and a sharp drop in political support for incumbents, public unrest erupted throughout sub-Saharan Africa (SSA) in 2024 (Peiris and Clément, 2008; Waddington et al., 2019). The World Bank’s (2025) Country Policy and Institutional Assessment (CPIA) report highlights growing dissatisfaction with public service quality across Sub Saharan Africa. This is particularly evident in infrastructure, health, education, and administration, where it has fuelled widespread unrest (Najimdeen, 2024). The report stresses that transparent and effective service delivery is key to rebuilding trust and promoting civic stability. The people in the area are not just statistics; they are individuals dealing with this predicament daily. They expressed increasing discontent with the standard of basic services, particularly in areas such as administrative responsiveness, infrastructure, education, and healthcare (Peiris and Clément, 2008; World Bank, 2025).

Studies have demonstrated that in Nigeria, corruption, inadequate monitoring, and low levels of citizen participation continue to result in subpar service delivery outcomes. This persists despite significant financial investments made in local governments (Adeyinka and Adewumi, 2023; Peiris and Clément, 2008; Masiya et al., 2019). According to Afrobarometer statistics (2025), the Kenyan population is actively engaged in civic participation but frequently feels ignored by elected authorities. Only 22% of respondents believe that local council members pay attention to their problems (Ibrahim et al., 2025). Shenga (Kamau et al., 2025) examines Mozambique’s experience to show how perceptions of corruption, media efficacy, and economic realities influence public assessments of government performance. The results clearly show that citizens remain emotionally invested in governance but have become disillusioned, losing faith in the system (Kamau et al., 2025). This loss of faith, not due to indifference but as a response to persistent dissatisfaction and unmet expectations, is a significant development in the country’s governance.

This study adds to ongoing debates about governance and subjective well-being in Africa. It calls for a policy shift that moves beyond delivery metrics and prioritises sociologically informed interventions. These interventions should foster transparency, community engagement, equitable access, and relational trust. Evidence from countries such as Nigeria, Kenya, Ghana, and Mozambique underscores the pressing need for reform (Adeyinka and Adewumi, 2023; Ang’anyo Onyango et al., 2024; Xatse and Naong, 2025). The World Bank’s CPIA report emphasises that public confidence depends on a government’s ability to transform resources into essential services efficiently (Shenga, 2019; Gumah and Aziabah, 2020). This capacity forms the bedrock of civic trust and nurtures a shared purpose between institutions and citizens. By embracing both the realities of service provision and the power of public perception, Gauteng can advance toward a governance model that upholds democratic values. It can also honour the everyday dignity of its citizens [Akokuwebe et al., 2023a; Gauteng City-Region Observatory (GCRO), 2023–2024]. The rationale for this study stems from the persistent disconnect between government policy frameworks, citizens’ lived realities, and ways in which the public experiences and interprets these policies. Despite the expansion of infrastructure and social programmes, public perception of governance remains marked by concerns over inefficiency, lack of transparency, and weak accountability (Zhou and Utete, 2023; World Bank, 2025). In Gauteng, growing dissatisfaction with basic services, particularly the decline in energy supply satisfaction from 78 to 42% over the past decade, signals a deeper crisis of legitimacy in governance [Akokuwebe et al., 2023a; Gauteng City-Region Observatory (GCRO), 2023–2024].

Utilising a comprehensive approach that combines both descriptive and inferential analyses, this study provides a nuanced understanding of how trust in governance varies across communities and social identities. It emphasises that satisfaction with service delivery is not just a material issue, but a crucial indicator of social inclusion and civic well-being. These findings, derived from a thorough and reliable study, help us comprehend how perceptions of government performance can influence and shape broader democratic attitudes and experiences. This study reflects a broader aim to critically examine the relationship between government performance and service delivery satisfaction in Gauteng. The analysis is grounded in robust empirical data and sociological insight. Specifically, it assesses the proportion of residents satisfied with service delivery at national, provincial, and municipal levels, explores associations between socio demographic characteristics and satisfaction, and identifies key predictors of perceptions of government performance. By anchoring this inquiry in both local realities and comparative perspectives, the study seeks to inform policy pathways that advance responsive governance and enhance the well-being of communities in Gauteng.

## Method

### Study area

The GCRO QoL Survey Round 5 (2017/18), 6 (2020/21), and 7 (2023/24) were conducted in Gauteng province, South Africa’s most populous, urbanised, and economically significant region. Gauteng comprises nine municipalities: the City of Johannesburg, City of Tshwane, City of Ekurhuleni, Emfuleni, Lesedi, Merafong City, Midvaal, Mogale City, and Rand West City. As the country’s industrial and financial hub, Gauteng is characterised by a high level of urbanisation, economic activity, and social diversity [Gauteng City-Region Observatory (GCRO), 2023–2024]. The province reflects South Africa’s linguistic and cultural plurality, being home to speakers of all eleven official languages. Demographically, it is predominantly Black African, with significant White and Coloured population groups, with the Indian/Asian population group being the smallest in size. Approximately 27.5% of the population is under the age of 15, while 9.7% are aged 60 and above [Gauteng City-Region Observatory (GCRO), 2023–2024]. Gauteng’s unique composition, shaped by historical mining activities and ongoing economic migration, makes it a microcosm of South Africa’s broader socio-political and economic landscape. Its diversity and urban infrastructure also makes it an ideal setting for assessing variations in citizen satisfaction with public service delivery and government performance [Gauteng City-Region Observatory (GCRO), 2023–2024].

### Study design and data source

This study utilised a cross-sectional design based on data from the GCRO QoL Survey Round 5 (2017/18), 6 (2020/21), and 7 (2023/24). A multi-stage, stratified cluster sampling technique was employed to select a representative sample of adults (aged 18 years and older) residing across Gauteng province [Gauteng City-Region Observatory (GCRO), 2023–2024]. The sampling frame was derived from the 2011 South African Population and Housing Census (2011 SA PHC), ensuring alignment with official enumeration boundaries [Gauteng City-Region Observatory (GCRO), 2023–2024]. In the first stage of sampling, enumeration areas (EAs) were identified within selected primary sampling units (PSUs), following the definitions outlined in the 2011 PHC [Gauteng City-Region Observatory (GCRO), 2023–2024]. In the second stage, systematic random sampling was used to catalogue and select households within each EA. All adults aged 18 years and above in the selected households were eligible to participate in the survey [Gauteng City-Region Observatory (GCRO), 2023–2024]. Round 5 (2017/18), 6 (2020/21), and 7 (2023/24) covered 24889, 13616, and 13795 respondents, respectively, during the survey periods, and is also the most recent and comprehensive iterations of the programme to date. To ensure proportional representation, sampling clusters were allocated to urban and rural strata within each municipality, according to population distributions recorded in the 2011 census. Primary sampling units were then proportionally assigned based on the number of households in these strata, providing a robust empirical foundation for inference [Gauteng City-Region Observatory (GCRO), 2023–2024]. Round 5 (2017/18), 6 (2020/21), and 7 (2023/24) survey was conducted in collaboration with the University of the Witwatersrand (Wits), the Gauteng Provincial Government (GPG), and various municipalities within the province [Gauteng City-Region Observatory (GCRO), 2023–2024]. The dataset includes information on a wide range of domains, including quality of life, socio-economic status, satisfaction with government services, psychosocial attitudes, and values. The GCRO’s QoL programme serves as a strategic national initiative to produce harmonised, policy relevant data for planning, monitoring, and evaluating development outcomes. These outcomes include progress toward the Sustainable Development Goals (SDGs) and other global commitments [Gauteng City-Region Observatory (GCRO), 2023–2024].

### Study population and sample size

The Round 5 (2017/18), 6 (2020/21), and 7 (2023/24) surveys employed a four stage multi-cluster sampling technique to randomly select respondents aged 18 years and older. A total of 24,889, 13,616, and 13,795 participants were selected, respectively, from two district municipalities, six local municipalities, and three metropolitan municipalities in Gauteng province. The sample was drawn from all 529 wards to ensure broad geographic and demographic representation. The QoL survey is a recurring household based study that captures data on residents’ satisfaction with service delivery. It also collects information on socio economic conditions, psychosocial attitudes, values, and other key characteristics. The survey instrument was developed by the GCRO in collaboration with academic experts, provincial stakeholders, and representatives from the three metropolitan municipalities. Data collection was conducted by trained fieldworkers using structured questionnaires.

### Measures

Data were collected by trained fieldworkers from the GCRO’s QoL Survey Round 5, 6 and 7. The survey instrument included the full questionnaire, comprising about 200 questions organised into 15 thematic modules. These covered a wide range of topics including demographics, housing, household services, migration, health, education, employment, community services, financial status, household assets, public participation and governance, perceived well-being, and overall quality of life. The specific variables included population group, sex, age, language, dwelling type, tenure, satisfaction and quality of housing, access to water, sanitation, refuse removal, energy sources, disability, sector of employment, amenities, transport, safety, crime, debts, income, social grants, and various household assets such as telephone, television, computer, radio, satellite TV, internet, car, bicycle, and fridge. Data were collected through structured questionnaires administered to randomly selected adult residents (men and women) within sampled households. While the full questionnaire encompasses diverse aspects of urban life, this study specifically focuses on citizen satisfaction with public service delivery. In this context, service performance is defined as the actual delivery of services by government institutions across various governance levels.

## Study variables

### Outcome variable

The primary outcome variable in this study is satisfaction with public service, serving as a proxy for assessing the performance of government institutions in service delivery. Satisfaction was measured through three survey items: (1) “How satisfied are you with the performance of the Gauteng Provincial Government?” (2) “How satisfied are you with the performance of the Local Municipality where you live?” and (3) “How satisfied are you with your local ward councillor?” Respondents rated their satisfaction using a five-point Likert scale: (1) very satisfied, (2) satisfied, (3) neither satisfied nor dissatisfied, (4) dissatisfied, and (5) very dissatisfied. For analytical purposes, responses were recoded into a binary variable: responses of “very satisfied” and “satisfied” were combined and coded as ‘1’ (satisfaction), while “dissatisfied” and “very dissatisfied” were combined and coded as ‘0’ (dissatisfaction). The neutral category, “neither satisfied nor dissatisfied,” was excluded from the analysis. This was done to ensure a clear binary classification and to enhance the interpretability and precision of the logistic regression model.

### Explanatory factors

The explanatory variables for this study were classified into demographic, socio-economic, and political factors, as well as participation measures. The socio-demographic factors are age, sex, and population group. The socio-economic factors are education, employed in the past 7 days, victim of crime in the past 12 months, sanitation (satisfaction with sanitation), and healthcare (satisfaction with healthcare). Also, sanitation was generated using three service delivery-related questions: (1) “How satisfied are you with the water you currently have access to?” (2) “How satisfied are you with the sewerage service you currently have access to?” and (3) “How satisfied are you with the rubbish removal service you currently have access to?”. The neutral category “neither satisfied nor dissatisfied” was excluded, while “dissatisfied” and “very dissatisfied” were combined and coded as ‘1’, and “very satisfied” and “satisfied” were combined and coded as ‘2’ (satisfaction). The political participation measures include the following: social participation, political participation, participating in IDP processes, municipality involves you in planning, registered voter, principles of Batho Pele^1^ (In general, do you think most government officials are doing their best to service the people according to the principles of Batho Pele?), asked for a bribe by a government official, protests in the neighbourhood, province of birth, and period. The variable period was generated by creating an iteration variable that represented each survey round (5 to 7). Each round was named pre lockdown (2017/18), during lockdown (2020/21), and post lockdown (2023/24). These explanatory factors were treated as covariates in the analysis. Detailed descriptions of each variable are provided and summarized in Table 1.

**Table 1:** Summary of variable categories and descriptions.

Variables	Categorization
Socio-demographic factors	
Age	1 = 18–29; 2 = 30–39; 3 = 40–49; 4 = 50–59; 5 = 60+
Sex	1 = Male; 2 = Female
Population group	1 = Black African; 2 = Coloured; 3 = Indian/Asian; 4 = White
Socio-economic factors	
Education	1 = Primary/Less; 2 = Incomplete; 3 = Matric; 4 = Tertiary
Employed in the past 7 days	1 = No; 2 = Yes
Victim of crime in the past 12 months	1 = No; 2 = Yes
Sanitation	1 = Dissatisfied; 2 = Satisfied
Healthcare	1 = Dissatisfied; 2 = Satisfied
Political participation measures	
Social participation	1 = No; 2 = Yes
Political participation	1 = No; 2 = Yes
Participating in IDP processes	1 = No; 2 = Yes
Municipality involves you in planning	1 = No; 2 = Yes
Registered voter	1 = No; 2 = Yes
Principles of Batho Pele	1 = No; 2 = Yes
Asked for a bribe by a government official	1 = No; 2 = Yes
Protests in the neighbourhood	1 = No; 2 = Yes
Province of birth	1 = Gauteng; 2 = Outside Gauteng; 3 = Outside South Africa
Period	1 = Pre lockdown; 2 = During lockdown; 3 = Post lockdown

Authors’ compilation, 2025.

### Data preparation and analysis

**Figure 1:**
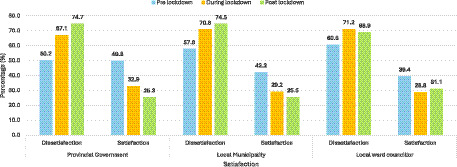
Public satisfaction and dissatisfaction with government entities across lockdown phases.

The study aimed to examine the socio-demographic, socio-economic, and political participation factors that influence residents’ satisfaction with government service delivery. This was assessed across various spheres of government among individuals aged 18 years and older. The demographic characteristics of the study population were stratified by governance level (Table 2). The distribution of respondents’ satisfaction levels was summarised using descriptive statistics (Figure 1). Trends in public perceptions of government performance before, during, and after lockdown were presented in a bar graph (Figure 1). To assess the strength and direction of the relationship between satisfaction and key explanatory variables, including demographic, economic, and political engagement indicators, Generalized Estimating Equations (GEE) regression analysis was conducted. The analysis was performed at a 5% level of significance (Tables 3, 4). GEE was selected because it accommodates repeated measures on the same individuals and accounts for within subject correlation. It also yields robust population averaged estimates (Peltzer, 2018; Capitine et al., 2021; Mahalingam et al., 2025). Furthermore, the GCRO employs a multistage sampling design, and multiple survey years were merged. This may have resulted in respondents being nested within the same clusters. The GEE assumes that observations between clusters are independent, while observations within clusters are equally correlated. This clustering makes GEE an appropriate analytical approach. The working correlation structure was selected based on model fit diagnostics. GEE also allows the use of all available observation pairs when data are intermittently missing. All data processing and statistical analyses were conducted using the statistical software STATA version 15. The dataset was weighted, and the weighting variables of each survey along with the “svy command” were applied to address over and under sampling biases. This also accounted for the complex survey design and improved the generalisability of the findings. Missing values were excluded from the analysis because the respective data points were not captured during the data collection phase. As a result, they did not exist within the dataset, making imputation or correction infeasible. Lastly, multicollinearity was checked using the “vif” command in the Stata software; the mean vif was 1.46.

**Table 2:** Respondent’s characteristics and government performance stratified by service delivery satisfaction.

Characteristics	Provincial government						Local municipality						Local ward councillor					
	Dissatisfied		Satisfied		χ^2^	*p* value	Dissatisfied		Satisfied		χ^2^	*p* value	Dissatisfied		Satisfied		χ^2^	*p* value
	Frequency	%	Frequency	%			Frequency	%	Frequency	%			Frequency	%	Frequency	%		
Age					23.80	0.00					35.25	0.00					117.08	0.00
18–29	3,294	62.7	1,963	37.3			3,575	68.0	1,682	32.0			3,638	69.2	1,619	30.8		
30–39	3,551	62.6	2,125	37.4			3,773	66.5	1,903	33.5			3,798	66.9	1,878	33.1		
40–49	2,895	63.1	1,693	36.9			3,087	67.3	1,501	32.7			3,040	66.3	1,548	33.7		
50–59	1,867	60.4	1,224	39.6			2,005	64.9	1,086	35.1			2,010	65.0	1,082	35.0		
60+	1,901	57.6	1,398	42.4			2,025	61.4	1,274	38.6			1,909	57.9	1,390	42.1		
**Sex**					0.76	0.38					2.58	0.11					0.12	0.73
Male	6,604	62.0	4,048	38.0			7,038	66.1	3,614	33.9			7,066	66.3	3,586	33.7		
Female	6,904	61.3	4,356	38.7			7,427	66.0	3,832	34.0			7,329	65.1	3,931	34.9		
Population group					23.92	0.00					478.09	0.00					1300.00	0.00
Black Africans	11,563	61.5	7,250	38.5			12,810	68.1	6,003	31.9			13,089	69.6	5,724	30.4		
Coloured people	492	69.0	221	31.0			495	69.5	217	30.5			472	66.2	241	33.8		
Indian/Asian people	241	63.4	139	36.6			195	51.3	185	48.7			169	44.3	212	55.7		
White people	1,212	60.4	793	39.6			965	48.1	1,040	51.9			666	33.2	1,339	66.8		
Education					124.69	0.00					180.96	0.00					413.80	0.00
Primary/less	1,294	51.8	1,204	48.2			1,574	63.0	925	37.0			1,686	67.5	812	32.5		
Incomplete Secondary	4,425	61.3	2,789	38.7			4,936	68.4	2,278	31.6			5,040	69.9	2,174	30.1		
Matric	4,763	65.4	2,524	34.6			5,078	69.7	2,209	30.3			5,025	69.0	2,262	31.0		
Tertiary	3,025	61.6	1,887	38.4			2,878	58.6	2,035	41.4			2,644	53.8	2,268	46.2		
Worked in the past 7 days					3.92	0.05					28.50	0.00					101.02	0.00
No	8,299	61.0	5,310	39.0			9,137	67.1	4,471	32.9			9,216	67.7	4,393	32.3		
Yes	5,209	62.7	3,094	37.3			5,328	64.2	2,975	35.8			5,179	62.4	3,124	37.6		
Victim of crime					121.66	0.00					153.56	0.00					110.53	0.00
No	10,243	59.9	6,870	40.1			10,969	64.1	6,143	35.9			10,969	64.1	6,144	35.9		
Yes	3,265	68.0	1,534	32.0			3,496	72.9	1,303	27.1			3,426	71.4	1,373	28.6		
Sanitation					892.90	0.00					1900.00	0.00					1500.00	0.00
Dissatisfied	6,702	72.5	2,542	27.5			7,551	81.7	1,694	18.3			7,401	80.1	1,843	19.9		
Satisfied	6,805	53.7	5,862	46.3			6,915	54.6	5,752	45.4			6,994	55.2	5,673	44.8		
Healthcare					649.81	0.00					1000.00	0.00					1100.00	0.00
Dissatisfied	5,516	74.6	1,882	25.4			5,951	80.4	1,448	19.6			5,940	80.3	1,458	19.7		
Satisfied	7,991	55.1	6,522	44.9			8,515	58.7	5,998	41.3			8,455	58.3	6,058	41.7		
Social participation					21.33	0.00					10.53	0.00					0.28	0.59
No	10,974	61.2	6,969	38.8			11,785	65.7	6,158	34.3			11,797	65.7	6,147	34.3		
Yes	2,533	63.8	1,435	36.2			2,680	67.6	1,288	32.4			2,598	65.5	1,370	34.5		
Political participation					21.58	0.00					2.87	0.09					1.28	0.26
No	12,489	62.2	7,577	37.8			13,301	66.3	6,764	33.7			13,210	65.8	6,856	34.2		
Yes	1,019	55.2	827	44.8			1,164	63.1	682	36.9			1,185	64.2	661	35.8		
Participating in IDP processes					2.98	0.08					14.96	0.00					0.58	0.45
No	12,327	61.4	7,738	38.6			13,171	65.6	6,893	34.4			13,185	65.7	6,879	34.3		
Yes	1,181	63.9	666	36.1			1,294	70.1	553	29.9			1,210	65.5	637	34.5		
Municipality involves you in planning					2200.00	0.00					2800.00	0.00					2300.00	0.00
Agree	2,166	39.1	3,381	60.9			2,233	40.2	3,315	59.8			2,293	41.3	3,254	58.7		
Neither	2,406	57.9	1,752	42.1			2,558	61.5	1,600	38.5			2,594	62.4	1,563	37.6		
Disagree	8,935	73.2	3,271	26.8			9,675	79.3	2,531	20.7			9,508	77.9	2,699	22.1		
Registered to vote					0.77	0.38					0.35	0.55					1.33	0.02
No	2,894	61.4	1,820	38.6			3,073	65.2	1,640	34.8			3,113	66.0	1,601	34.0		
Yes	10,613	61.7	6,584	38.3			11,392	66.2	5,806	33.8			11,282	65.6	5,916	34.4		
Principles of Batho Pele					2400.00	0.00					2000.00	0.00					1500.00	**0.00**
No	11,275	71.7	4,460	28.3			11,808	75.0	3,927	25.0			11,573	73.5	4,162	26.5		
Yes	2,232	36.1	3,944	63.9			2,657	43.0	3,519	57.0			2,822	45.7	3,354	54.3		
Asked for a bribe					25.30	0.00					0.00	0.99						
No	11,702	61.1	7,450	38.9			12,662	66.1	6,490	33.9			12,605	65.8	6,547	34.2	0.39	0.53
Yes	1,805	65.4	954	34.6			1,803	65.4	956	34.6			1,790	64.9	970	35.1		
Protests in the neighborhood					233.25	0.00					631.74	0.00						
No	8,119	57.9	5,906	42.1			8,464	60.3	5,561	39.7			8,319	59.3	5,706	40.7	747.52	0.00
Yes	5,389	68.3	2,498	31.7			6,001	76.1	1,885	23.9			6,076	77.0	1,811	23.0		
Province of birth					201.97	0.00					245.85	0.00					236.39	0.00
Gauteng	8,127	63.5	4,663	36.5			8,609	67.3	4,181	32.7			8,533	66.7	4,258	33.3		
Outside Gauteng	4,859	61.8	3,010	38.2			5,293	67.3	2,576	32.7			5,287	67.2	2,582	32.8		
Outside SA	521	41.6	731	58.4			563	44.9	689	55.1			575	45.9	677	54.1		
Wave					1200.00	0.00					524.98	0.00					192.46	0.00
Pre lockdown	4,988	50.2	4,958	49.8			5,749	57.8	4,197	42.2			6,027	60.6	3,919	39.4		
Lockdown	3,675	67.1	1,801	32.9			3,879	70.8	1,597	29.2			3,896	71.2	1,580	28.8		
Post lockdown	4,845	74.7	1,645	25.3			4,838	74.5	1,652	25.5			4,472	68.9	2,018	31.1		

*p < 0.05; **p < 0.01; ***p < 0.001.

**Table 3:** Unadjusted GEE regression analysis of satisfaction with government service delivery across governance levels.

Characteristics	Provincial government				Local municipality				Local ward councillor			
	UOR	P|Z|	95% CI		UOR	P|Z|	95% CI		UOR	P|Z|	95% CI	
Age												
18–29												
30–39	1.01	0.744	0.937	1.095	1.08	0.049	1.000	1.174	1.16	0.000	1.066	1.252
40–49	1.01	0.859	0.928	1.094	1.10	0.033	1.007	1.192	1.25	0.000	1.153	1.364
50–59	1.03	0.578	0.937	1.123	1.13	0.008	1.034	1.244	1.27	0.000	1.161	1.396
60+	1.21	0.000	1.108	1.320	1.30	0.000	1.191	1.424	1.61	0.000	1.473	1.760
Sex												
Male												
Female	1.02	0.389	0.970	1.081	0.96	0.108	0.905	1.010	0.99	0.728	0.937	1.046
Population group												
Black Africans												
Coloured people	0.69	0.000	0.594	0.803	0.92	0.294	0.794	1.072	1.16	0.052	0.999	1.338
Indian/Asian people	1.07	0.555	0.859	1.328	2.10	0.000	1.692	2.598	2.81	0.000	2.265	3.493
White people	0.99	0.796	0.907	1.078	2.44	0.000	2.239	2.656	4.64	0.000	4.239	5.086
Education												
Primary/less												
Incomplete Secondary	0.70	0.000	0.643	0.771	0.83	0.000	0.753	0.907	0.87	0.003	0.787	0.952
Matric	0.60	0.000	0.545	0.654	0.82	0.000	0.747	0.900	0.97	0.521	0.882	1.065
Tertiary	0.69	0.000	0.626	0.756	1.28	0.000	1.161	1.408	1.76	0.000	1.593	1.935
Worked in the past 7 days												
No												
Yes	0.95	0.048	0.894	1.000	1.17	0.000	1.102	1.234	1.33	0.000	1.259	1.409
Victim of crime												
No												
Yes	0.68	0.000	0.639	0.731	0.64	0.000	0.600	0.690	0.69	0.000	0.646	0.742
Sanitation												
Dissatisfied												
Satisfied	2.40	0.000	2.266	2.546	4.03	0.000	3.781	4.297	3.32	0.000	3.119	3.527
Healthcare												
Dissatisfied												
Satisfied	2.23	0.000	2.094	2.371	2.90	0.000	2.713	3.099	3.03	0.000	2.839	3.244
Social participation												
No												
Yes	0.84	0.000	0.786	0.907	0.89	0.001	0.824	0.953	0.98	0.598	0.913	1.054
Political participation												
No												
Yes	1.25	0.000	1.139	1.377	1.09	0.088	0.988	1.198	1.06	0.251	0.961	1.166
Participating in IDP processes												
No												
Yes	0.92	0.085	0.833	1.012	0.82	0.000	0.741	0.907	0.96	0.446	0.873	1.062
Municipality involves in planning												
Agree												
Neither	0.43	0.000	0.393	0.463	0.40	0.000	0.373	0.439	0.43	0.000	0.396	0.466
Disagree	0.22	0.000	0.202	0.231	0.17	0.000	0.158	0.181	0.20	0.000	0.189	0.216
Registered to vote												
No												
Yes	0.97	0.379	0.908	1.037	0.98	0.552	0.916	1.048	1.04	0.252	0.972	1.113
Principles of Batho Pele												
No												
Yes	4.53	0.000	4.262	4.825	3.86	0.000	3.630	4.101	3.20	0.000	3.009	3.395
Asked for a bribe												
No												
Yes	0.81	0.000	0.741	0.876	1.00	0.990	0.920	1.088	1.03	0.532	0.945	1.116
Protests in the neighborhood												
No												
Yes	0.63	0.000	0.597	0.672	0.45	0.000	0.425	0.482	0.42	0.000	0.398	0.450
Province of birth												
Gauteng												
Outside Gauteng	1.09	0.005	1.026	1.151	1.04	0.230	0.977	1.100	1.01	0.830	0.949	1.068
Outside SA	2.23	0.000	1.995	2.502	2.40	0.000	2.143	2.684	2.34	0.000	2.093	2.622
Period												
Pre COVID												
During COVID	0.48	0.000	0.451	0.517	0.57	0.000	0.533	0.611	0.64	0.000	0.599	0.687
Post COVID	0.33	0.000	0.305	0.350	0.49	0.000	0.459	0.526	0.73	0.000	0.680	0.774

Significant at ***p < 0.001, **p < 0.01, *p < 0.05; CI = Confidence interval; Ref = reference category; OR = Odd ratio.

**Table 4:** Adjusted GEE regression analysis of satisfaction with government service delivery across governance levels.

Characteristics	Provincial government				Local municipality				Local ward councillor			
	AOR	P|Z|	95% C. I.		AOR	P|Z|	95% C. I.		AOR	P|Z|	95% C. I.	
Age												
18–29												
30–39	0.90	0.026	0.820	0.987	0.91	0.071	0.830	1.008	0.97	0.585	0.886	1.070
40–49	0.87	0.007	0.789	0.963	0.87	0.009	0.782	0.965	1.00	0.986	0.902	1.107
50–59	0.89	0.045	0.796	0.997	0.91	0.105	0.807	1.020	1.01	0.846	0.901	1.136
60+	0.97	0.569	0.859	1.087	0.91	0.118	0.802	1.025	1.17	0.009	1.042	1.322
Sex												
Male												
Female	1.03	0.418	0.963	1.094	1.00	0.883	0.931	1.063	1.08	0.020	1.012	1.153
Population group												
Black Africans												
Coloured people	0.74	0.000	0.628	0.878	0.97	0.743	0.817	1.155	1.25	0.009	1.057	1.474
Indian/Asian people	0.99	0.932	0.780	1.256	1.71	0.000	1.321	2.204	2.14	0.000	1.646	2.789
White people	0.83	0.001	0.748	0.928	1.98	0.000	1.765	2.210	3.71	0.000	3.303	4.168
Education												
Primary/less												
Incomplete Secondary	0.78	0.000	0.699	0.871	0.88	0.031	0.786	0.989	0.95	0.402	0.852	1.066
Matric	0.65	0.000	0.575	0.727	0.79	0.000	0.702	0.896	1.00	0.980	0.889	1.128
Tertiary	0.62	0.000	0.547	0.702	0.85	0.014	0.747	0.968	1.15	0.032	1.012	1.304
Worked in the past 7 days												
No												
Yes	1.01	0.696	0.946	1.087	1.09	0.025	1.011	1.167	1.19	0.000	1.108	1.277
Victim of crime												
No												
Yes	0.79	0.000	0.729	0.854	0.77	0.000	0.710	0.839	0.84	0.000	0.771	0.905
Sanitation												
Dissatisfied												
Satisfied	1.81	0.000	1.694	1.942	2.94	0.000	2.730	3.162	2.24	0.000	2.092	2.408
Healthcare												
Dissatisfied												
Satisfied	1.77	0.000	1.649	1.906	1.84	0.000	1.706	1.994	1.73	0.000	1.600	1.863
Social participation												
No												
Yes	0.94	0.122	0.860	1.018	0.94	0.196	0.864	1.030	1.00	0.995	0.918	1.090
Political participation												
No												
Yes	1.30	0.000	1.162	1.456	1.22	0.001	1.082	1.372	1.20	0.003	1.062	1.349
Participating in IDP processes												
No												
Yes	1.31	0.000	1.169	1.469	1.18	0.006	1.049	1.336	1.39	0.000	1.234	1.565
Municipality involves you in planning												
Agree												
Neither	0.55	0.000	0.501	0.598	0.48	0.000	0.441	0.528	0.50	0.000	0.453	0.541
Disagree	0.30	0.000	0.281	0.325	0.24	0.000	0.220	0.256	0.27	0.000	0.255	0.295
Registered to vote												
No												
Yes	1.02	0.733	0.929	1.110	0.98	0.720	0.896	1.079	0.98	0.594	0.890	1.069
Principles of Batho Pele												
No												
Yes	2.80	0.000	2.617	3.002	2.55	0.000	2.375	2.739	2.36	0.000	2.199	2.532
Asked for a bribe												
No												
Yes	0.83	0.000	0.752	0.914	0.97	0.498	0.873	1.068	0.93	0.183	0.846	1.032
Protests in the neighborhood												
No												
Yes	0.77	0.000	0.721	0.829	0.64	0.000	0.597	0.693	0.64	0.000	0.592	0.685
Province of birth												
Gauteng												
Outside Gauteng	1.12	0.001	1.045	1.195	1.17	0.000	1.091	1.257	1.16	0.000	1.079	1.240
Outside SA	2.25	0.000	1.956	2.581	2.40	0.000	2.077	2.771	2.37	0.000	2.047	2.742
Period												
Pre COVID												
During COVID	0.55	0.000	0.510	0.595	0.65	0.000	0.596	0.704	0.68	0.000	0.627	0.739
Post COVID	0.37	0.000	0.341	0.401	0.58	0.000	0.530	0.628	0.82	0.000	0.756	0.892

Significant at ***p < 0.000, **p < 0.01, *p < 0.05; CI = Confidence interval; Ref = reference category; OR = Odd ratio.

### Ethical considerations

The GCRO QoL Survey Round 5 (2017/18), 6 (2020/21), and 7 (2023/24) received ethical clearance from the Human Research Ethics Committee (HREC) (non-medical) at the University of the Witwatersrand, South Africa. Verbal consent was obtained from all respondents aged 18 years and older, after they were fully informed about the voluntary nature of the study, and assured of confidentiality and anonymity. To confirm their participation, each respondent was issued a signed hardcopy receipt. Fieldworkers also carried official documentation, including a letter from the Gauteng Premier and the ethics clearance certificate, which they presented when necessary to facilitate access to fieldwork locations or to clarify the purpose of the study.

## Results

The final sample sizes used for the analysis for each governance level were Gauteng provincial government (*n* = 9,946), local municipality (*n* = 9,946), and local ward councillor (*n* = 9,946). These were further disaggregated into satisfied and dissatisfied respondents for each governance level. For the Gauteng provincial government, satisfied (*n* = 4,958) and dissatisfied (*n* = 4,988); for the local municipality, satisfied (*n* = 4,197) and dissatisfied (*n* = 5,749); and for the local ward councillor, satisfied (*n* = 3,919) and dissatisfied (*n* = 6,027). These stratified variables served as the outcome variable in the logistic regression analysis across the governance levels.

### Trends in public perceptions of government performance before, during, and after lockdown

Figure 1 illustrates shifting public sentiment toward government performance across lockdown phases. The data show a consistent increase in dissatisfaction over time, accompanied by declining satisfaction across all spheres. For the provincial government, dissatisfaction rose from 50.2% before lockdown to 67.1% during lockdown, peaking at 74.7% post lockdown (Figure 1). Similarly, dissatisfaction with local municipalities increased from 57.8% pre lockdown to 70.8% during lockdown, reaching 74.5% post lockdown. Among local ward councillors, dissatisfaction peaked during lockdown at 71.2% but showed a slight decline post lockdown to 68.9%, while satisfaction increased modestly from 28.8 to 31.1%.

### Age-disaggregated trends in satisfaction and dissatisfaction with provincial, municipal, and ward councillor performance across lockdown phases

**Figure 2:**
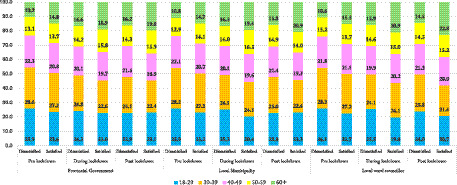
Age-based trends in satisfaction with government performance across lockdown phases.

Figure 2 illustrates age disaggregated trends in public satisfaction and dissatisfaction. Across all government tiers and time phases, younger respondents (18–29 and 30–39 years) consistently reported higher levels of dissatisfaction, while older respondents, particularly those aged 60 years and above, expressed comparatively greater satisfaction. This pattern suggests generational differences in expectations and levels of institutional trust between younger and older age groups.

### Sex based patterns in satisfaction with government performance across lockdown phases

**Figure 3:**
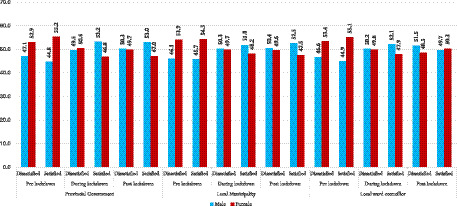
Sex-based patterns of satisfaction and dissatisfaction with government performance across lockdown phases.

Figure 3 compares male and female satisfaction and dissatisfaction with government performance across lockdown phases. Before lockdown, females reported slightly higher dissatisfaction than males across all three government tiers, while also expressing marginally higher satisfaction, particularly with ward councillors (55.1% among females versus 44.9% among males). During lockdown, male satisfaction increased slightly across all entities and surpassed that of females, especially for the provincial government (53.2% among males versus 46.8% among females) (Figure 3). After lockdown, male satisfaction remained higher for provincial and municipal governments, while female satisfaction slightly exceeded that of males among ward councillors (50.3% versus 49.7%). Overall, the findings suggest that perceptions of service delivery and governance were broadly similar across male and female respondents.

### Determinants of government performance and service delivery satisfaction

The Chi square test indicates statistically significant associations across most variables (Table 2). Dissatisfaction was consistently high across age groups, with younger respondents (18–29 years) reporting the highest levels, while older respondents (60^+^) showed greater satisfaction, particularly with national (42.4%) and ward councillors (42.1%). Sex differences were not significant (*p* > 0.05). Population group differences were pronounced: Black African respondents reported the highest dissatisfaction (over 60% across tiers), while White and Indian/Asian respondents showed higher satisfaction, especially with ward councillors (66.8% among White people; 55.7% among Indians/Asians people) (*p* < 0.001) (Table 2). Dissatisfaction with government increased with higher education and unemployment, with over 70 to 80% dissatisfaction reported among those experiencing poor sanitation and healthcare services, and among crime victims. In contrast, satisfaction was higher among those engaged in political processes and those who perceived municipal inclusiveness in planning (58–60%), as well as among respondents who believed officials upheld Batho Pele principles (54–64%), while over 70% of those with negative perceptions remained dissatisfied. Living in protest affected areas and the COVID 19 lockdown further intensified dissatisfaction across all tiers, whereas respondents born outside South Africa reported comparatively higher satisfaction. Overall, perceptions were strongly shaped by service delivery quality, participatory governance, and socio demographic inequalities.

### Unadjusted generalized estimating equations (GEE) regression analysis

Table 3 shows the unadjusted GEE results across the three governance levels. Older adults reported progressively higher satisfaction than those aged 18–29, with the strongest effects among respondents aged 60+ across provincial (UOR 1.21, *p* < 0.001), municipal (UOR 1.30, *p* < 0.001), and ward levels (UOR 1.61, *p* < 0.001). Sex differences were minimal. Population group differences were notable: Coloured respondents were less satisfied provincially, while Indian/Asian and White respondents showed substantially higher satisfaction at municipal and ward levels, especially White respondents (UOR 4.64, *p* < 0.001 at ward level) (Table 3). Education showed mixed effects. Lower education levels were associated with reduced satisfaction provincially and municipally, whereas tertiary education predicted higher satisfaction at municipal (UOR 1.28, *p* < 0.001) and ward levels (UOR 1.76, *p* < 0.001). Recent employment increased satisfaction municipally and at ward level. Victims of crime consistently reported lower satisfaction across all levels. Service delivery indicators were among the strongest predictors. Satisfaction with sanitation and healthcare services significantly increased satisfaction across all governance levels, with the largest effects at municipal and ward levels (Table 3). Social participation reduced satisfaction provincially and municipally, while political participation increased satisfaction only provincially. Participation in IDP processes lowered satisfaction municipally. Perceptions of municipal involvement in planning were strongly associated with satisfaction; respondents who disagreed had markedly lower odds across all levels. Batho Pele adherence was one of the strongest positive predictors across provincial (UOR 4.53, *p* < 0.001), municipal (UOR 3.86, *p* < 0.001), and ward levels (UOR 3.20, *p* < 0.001) (Table 3). Being asked for a bribe was found to reduce satisfaction at the provincial level, while protests in the neighbourhood significantly lowered satisfaction across all levels. Respondents born outside South Africa reported substantially higher satisfaction across all the governance levels. Satisfaction declined significantly during and after the COVID-19 period, with the sharpest drop observed at the provincial level in the post-COVID period (UOR 0.33, *p* < 0.001) (Table 3).

### Adjusted generalized estimating equations (GEE) regression analysis

The adjusted GEE analysis identified several significant predictors of satisfaction across provincial, municipal, and ward councillor levels (Table 4). Older age groups generally showed lower satisfaction at provincial and municipal levels, while adults aged 60 years and above were more satisfied with ward councillors. Population group differences were marked, with Coloured respondents reported less satisfied at the provincial level but more satisfied with the ward councillors, and Indian/Asian and White respondents reported higher satisfaction at the municipal and ward levels. Respondents with higher education was found associated with lower satisfaction at both at the provincial and municipal levels, but tertiary education increased satisfaction with ward councillors (Table 4). Victims of crime consistently reported lower satisfaction across all governance levels. Satisfaction with sanitation and healthcare services strongly predicted higher satisfaction, with the largest effects observed at municipal and ward levels. Political participation and involvement in integrated development planning (IDP) processes significantly increased satisfaction across all levels. Respondents who perceived municipalities as uninvolved in planning had substantially reported lower satisfaction (Table 4). Principles of Batho Pele was a strong positive predictor across all governance levels, while exposure to bribery was found to reduce satisfaction at the provincial level. Respondents living in areas with protests significantly reported lowered satisfaction, and respondents born outside Gauteng or outside South Africa were found to be more satisfied across all governance levels. Satisfaction declined significantly during and after the COVID-19 period compared with the pre-COVID period (Table 4).

## Discussion

This study assessed public satisfaction and dissatisfaction with government performance and service delivery across three governance levels (provincial government, local municipality, and local ward councillor), using the GCRO QoL Survey Round 5 (2017/18), 6 (2020/21), and 7 (2023/24) in Gauteng Province, South Africa. The findings consistently revealed widespread dissatisfaction, with notable variations across age, population group, education, employment status, and civic participation. Logistic regression analyses further identified socio demographic, perceptual, and participatory predictors of satisfaction. These findings underscore the complex interplay between structural inequalities, governance practices, and public trust (Zhou and Utete, 2023). Three overarching themes emerged from the findings. These include: (1) the intensification of dissatisfaction during and after lockdown, (2) generational and population group disparities in perceptions of government performance, and the (3) critical role of accountability, communication, and trust in shaping satisfaction with service delivery. These themes highlight that perceptions of government performance are deeply intertwined with both structural inequalities and subjective experiences. These include population group, socio-economic status, political engagement, exposure to crime, and institutional trust (Schoeman and Chakwizira, 2023; Peiris and Clément, 2008).

The descriptive analyses (Figures 1–3) demonstrated a clear trajectory of declining satisfaction and rising dissatisfaction across all governance tiers during the lockdown. Only modest recovery was observed post lockdown at the ward level. Provincial and municipal governments experienced the steepest declines. This suggests that citizens perceived their responses to the pandemic as inadequate or misaligned with community needs. Ward councillors, however, showed a slight rebound in satisfaction post-lockdown, particularly among older respondents. This may reflect the relative proximity and visibility of ward-level representatives, who are often more directly engaged in community problem-solving (Akokuwebe et al., 2025; Ang’anyo Onyango et al., 2024). These trends underscore the fragility of public confidence during crises. They reveal the extent to which government legitimacy is contingent on responsiveness, transparency, and effective service delivery in times of heightened vulnerability [Gauteng City-Region Observatory (GCRO), 2023–2024; Schoeman and Chakwizira, 2023].

Age emerged as a significant determinant of perceptions. Younger respondents (18–29 and 30–39 years) consistently reported the highest dissatisfaction across all tiers and phases, while older respondents (60^+^) expressed comparatively greater satisfaction. This generational divide suggests that younger cohorts may hold higher expectations of government performance or may be more critical. This may be due to their exposure to precarious employment, limited opportunities, and heightened socio economic vulnerabilities during the pandemic (Masiya et al., 2019; Kamau et al., 2025). Sex differences, by contrast, were minimal. Although females reported slightly higher dissatisfaction pre-lockdown and marginally higher satisfaction with ward councillors, overall patterns remained balanced between males and females. This finding suggests that dissatisfaction with government performance was broadly shared across genders. It reinforces the notion that structural and service delivery factors outweighed gendered experiences in shaping perceptions (World Bank, 2025; Ibrahim et al., 2025).

Table 2 highlighted the strong influence of race, education, and employment on satisfaction. Africans reported the highest dissatisfaction across tiers, while White and Indian/Asian respondents expressed comparatively higher satisfaction, particularly with ward councillors. These disparities may reflect historical inequalities in service delivery, differential access to resources, and varying expectations of government performance (Flavin, 2024; Zhou and Utete, 2023). Similar patterns have been noted in Namibia and Botswana, where population group and ethnicity intersect with access to political networks and patronage (Zerihun and Mashingo, 2022; Bratton and Logan, 2006; Good, 2016). These findings reinforce that perceptions of fairness and satisfaction with public institutions are strongly mediated by citizens’ structural access to political power and representation (Lamsal and Gupta, 2022; Adeyinka and Adewumi, 2023). Education was inversely associated with satisfaction, with matric and tertiary respondents reporting greater dissatisfaction compared to those with primary education or less. This suggests that more educated citizens may hold governments to higher standards, or may be more aware of governance shortcomings (Ibrahim et al., 2025; Shenga, 2019). Comparable findings in Kenya and Uganda show that higher educational attainment is linked to lower tolerance for corruption and administrative failures (Waddington et al., 2019; Melber, 2020). Employment status also mattered: unemployed respondents reported significantly lower satisfaction, particularly at municipal and ward levels, underscoring the link between economic precarity and perceptions of governance at all levels. Service delivery experiences were among the strongest predictors. Dissatisfaction with sanitation and healthcare was closely tied to dissatisfaction with government performance, while satisfaction with these services correlated with higher trust. These findings highlight the centrality of basic service provision in shaping public confidence. Moreover, experiences of crime significantly reduced satisfaction with provincial and municipal governments, reflecting the role of personal security in shaping trust (Male and Amalo, 2024; Cheeseman et al., 2021). Living in protest-affected areas was strongly associated with dissatisfaction across all tiers, suggesting that protest activity both reflects and reinforces discontent.

Civic engagement emerged as a critical determinant of satisfaction. Respondents who reported political participation or felt included in municipal planning expressed significantly higher satisfaction, while those excluded reported very high dissatisfaction. Principles of Batho Pele was strongly associated with satisfaction across all tiers, underscoring the importance of accountability, transparency, and citizen-centered governance (Mojapelo et al., 2021; Mabunda, 2024; Molobela, 2024). Logistic regression analyses (Table 3) reinforced these findings. Principles of Batho Pele nearly doubled the odds of satisfaction at provincial and municipal levels, while intention to vote in the 2024 elections positively influenced satisfaction at provincial and ward levels. Conversely, negative experiences and perceptions consistently reduced satisfaction across all governance levels (Good, 2016; Melber, 2020). Victims of crime, respondents exposed to bribery, and those living in areas with protests reported markedly lower odds of satisfaction, underscoring how insecurity and corruption erode public confidence (Cheeseman et al., 2021; Mabunda, 2024). Similarly, disagreement with municipal involvement in planning was strongly associated with dissatisfaction, highlighting the importance of participatory governance. The sharp decline in satisfaction during and after the COVID-19 period further illustrates how broader contextual shocks undermine trust in government performance. This resonates with findings across the continent, including Malawi, Nigeria, and Tunisia. In these contexts, declining trust in political elites is frequently associated with withdrawal from political processes and diminished public morale (Masiya et al., 2019; Logan and Cho, 2009).

The Gauteng findings mirror broader African experiences. In Botswana, ethnic majoritarianism has fostered patronage based politics, while in Namibia, ethnic favouritism has shaped political appointments and service delivery (Zerihun and Mashingo, 2022; Bratton and Logan, 2006; Good, 2016). In Ghana and Senegal, civic education campaigns have boosted citizens’ trust and satisfaction with public institutions (Lamsal and Gupta, 2022; Mattes and Moreno, 2018). Conversely, declining trust in leadership in Nigeria and Tunisia has undermined satisfaction with governance (Akokuwebe, 2017; Abioro, 2021; Whitfield, 2009). These comparative insights reinforce that dissatisfaction with government service delivery is not unique to South Africa. It reflects broader challenges of equity, accountability, and legitimacy across African democracies. The findings carry significant implications for governance and policy. Improving basic service delivery, particularly sanitation and healthcare, emerges as essential for rebuilding public trust [Gauteng City-Region Observatory (GCRO), 2023–2024; Schoeman and Chakwizira, 2023]. At the same time, targeted engagement strategies are required to address generational dissatisfaction, as younger cohorts consistently reported the lowest levels of satisfaction (Shenga, 2019; Gumah and Aziabah, 2020). Strengthening accountability mechanisms, including Batho Pele principles and participatory planning, can further enhance legitimacy and foster greater confidence in government institutions (Yerkes and Muasher, 2017; Rulashe and Ijeoma, 2022). Equally important is leadership credibility and communication, since distrust in government leaders was the most powerful predictor of dissatisfaction (Abioro, 2021; Rulashe and Ijeoma, 2022). The findings highlight that citizen satisfaction is inseparable from the broader dynamics of equity, participation, and integrity in governance. They underscore how perceptions of service delivery reflect deeper societal expectations of democratic accountability. Ultimately, fostering citizen satisfaction requires not only efficient service delivery but also a governance framework that consistently upholds fairness, inclusivity, and trust.

### Sociological insights on public satisfaction with governance

Public satisfaction with governance is socially produced and reflects how citizens are positioned within broader structures of inequality. Yet, it does not function as a simple assessment of service delivery performance. The findings show that racialised, educational, and use socio-economic inequalities systematically shape perceptions of government. This indicates that dissatisfaction is patterned by differential exposure to disadvantage and varying expectations of the state. Institutional trust functions as key mediating mechanism, where perceptions of leadership integrity, fairness, and responsiveness significantly shape levels of satisfaction. This shows that legitimacy is socially constructed through perceived state behaviour and often outweighs service delivery outcomes alone. Governance is therefore evaluated through principles of procedural justice, where transparency, accountability, and inclusion matter as much as tangible service delivery. In addition, satisfaction is strengthened through communicative and participatory governance. Citizens who feel heard, included, and recognised report higher levels of trust, underscoring the co-production of legitimacy through everyday state citizen interactions. Satisfaction reflects the intersection of material conditions and symbolic experiences of governance, where both inequality and recognition shape how the state is judged.

### Strengths and limitations of the study

#### Strengths

This study provides valuable insights into public satisfaction with government service delivery across different levels of governance in South Africa by combining descriptive and inferential analyses. The use of a large, representative dataset from the GCRO is a major strength, as it enabled nuanced disaggregation by race, education, employment, and political engagement. This granularity allowed for a deeper understanding of how structural inequalities and socio-demographic characteristics shape perceptions of government performance. It also captures the intersectional dynamics of satisfaction and dissatisfaction across diverse social groups. The inclusion of logistic regression further strengthened the analysis by identifying significant predictors of satisfaction while controlling for confounding variables. This enhanced the robustness and credibility of the findings. By linking service delivery outcomes to broader socio demographic and perceptual factors, the study contributes meaningfully to debates on governance, accountability, and institutional trust in South Africa. It offers a solid foundation for both policy development and sociological research.

#### Limitations

Despite the strengths of this study, several limitations must be acknowledged. The cross-sectional design restricts the ability to establish causal relationships between variables, meaning that observed associations cannot be interpreted as direct cause-and-effect. Reliance on self-reported data introduces the possibility of bias, as responses may be influenced by social desirability or recall inaccuracies. The focus on Gauteng Province offers rich insights into one of South Africa’s most dynamic regions. However, it limits the generalisability of the findings to other provinces or to the broader African context. Furthermore, the absence of qualitative perspectives constrains the ability to explore the deeper motivations and lived experiences behind satisfaction or dissatisfaction. These perspectives could have enriched the interpretation of the quantitative results.

### Policy implications for South Africa

Findings indicate that declining public trust is structurally driven and closely linked to persistent service delivery failures. This suggests that technical improvements alone are insufficient without addressing underlying governance deficits. The concentration of dissatisfaction at higher governance levels points to a legitimacy gap. This gap is reinforced by unequal access to basic services, particularly in marginalised communities. Socio demographic patterns reflect entrenched inequalities. Generational, gender, and population group differences show that satisfaction is shaped by unequal exposure to economic exclusion and historical disadvantage. These patterns imply that policy responses must move beyond uniform interventions. They require equity sensitive and targeted strategies that address uneven lived realities. The relative improvement in perceptions of ward level governance suggests that proximity, responsiveness, and communicative engagement are critical for rebuilding institutional trust. Strengthening legitimacy therefore requires ethical leadership and anti-corruption enforcement. It also requires sustained participatory governance and accountability mechanisms. Improving satisfaction depends on aligning service delivery with equity, trust building, and inclusive governance practices.

### Future research directions

Building on these findings, future research should adopt longitudinal designs to track changes in satisfaction over time. This would better capture causal relationships between socio demographic factors, service delivery experiences, and institutional trust. Incorporating qualitative approaches would also enrich understanding by uncovering the lived experiences and motivations behind satisfaction or dissatisfaction. This would offer deeper insights into how citizens interpret government performance. Expanding the scope beyond Gauteng to include other provinces and comparative African contexts would strengthen generalisability. It would also situate South Africa’s patterns within broader regional debates on governance and public trust. Hence, these directions can advance the sociology of satisfaction by integrating quantitative rigour with qualitative depth and comparative perspectives.

## Conclusion and recommendations

Dissatisfaction with government performance and service delivery in Gauteng is widespread, shaped by socio-demographic factors, service experiences, civic participation, and trust in leadership. Dissatisfaction intensified during lockdown and remained high afterward, particularly at the provincial and municipal levels, while ward councillors experienced modest recovery, suggesting that localised governance may be better positioned to rebuild trust. Generational divides were evident, with younger cohorts consistently more dissatisfied than older respondents, while differences across sex were minimal, indicating that dissatisfaction is driven less by gender and more by broader structural challenges in governance and service delivery. Population group differences remain decisive in shaping perceptions: Black African respondents reported the highest levels of dissatisfaction across governance tiers, underscoring the enduring impact of structural inequities and the urgent need for equity-driven interventions in South Africa. Crime and safety concerns, coupled with weak municipal communication, further eroded confidence, highlighting the critical importance of security and transparency in restoring trust. Restoring public confidence requires more than improved service quality; it calls for institutional reform, equitable engagement, and credible leadership. Policymakers must prioritise sanitation and healthcare, reinforce accountability mechanisms, uphold Batho Pele principles, and advance participatory governance. Strengthening communication between government and communities, addressing inequalities rooted in race, education, and employment, and ensuring ethical and transparent leadership are central to rebuilding institutional trust. These actions are critical for improving governance legitimacy and enhancing citizen satisfaction across all tiers of governance.

## Data Availability

The original contributions presented in the study are included in the article/supplementary material, further inquiries can be directed to the corresponding author.

## Footnotes

1 Principles of Batho Pele directly imply putting people first, putting people, and their concerns first, while providing public services with an emphasis on efficiency, effectiveness and sustainability.
